# An Experimental Essay on the Relative Physiological and Medicinal Properties of Iodine and Its Compounds

**Published:** 1838-01

**Authors:** 


### Art. XI.

*An Experimental Essay on the relative Physiological and Medicinal Properties of Iodine and its Compounds, being the Harveian Prize Dissertation for* 1837. By Charles Cogswell, a.b. m.d.—*Edinburgh*, 1837. 8vo. pp. 179.

This is a work of considerable labour and research, and well merits the honour of being crowned with the Harveian Prize. It comprehends an excellent digest of the physiological and therapeutic effects of iodine and its compounds, derived from the most authentic sources. As the author himself states, where a blank seemed to exist he has sometimes attempted to supply the deficiency; where statements were found to clash, it has been his endeavour to shew in what manner they might be reconciled; and in doubtful points, although he has carefully examined the observations of other men, yet he has trusted only to his own. The whole is executed in a methodical and distinct manner, and displays much of that sober spirit of induction, which may be said to characterize the school to which he belongs.

The medical properties of iodine, as is well known, were first brought into notice by Dr. Coindet of Geneva, from the idea having suggested itself to him, that, the ashes of the *Fucus vesiculosus*, long celebrated for the cure of bronchocele and other glandular enlargements, might owe their specific virtues to the possession of this ingredient. This was in a memoir presented to the Helvetic Society in August 1820. The wonderful success which attended his treatment of the endemic malady of his country, goitre, at length enabled him to triumph over the professional opposition and even malice with which the first communication of his valuable discovery was so strongly visited.

"The wide extent of diffusion in which iodine now appears to exist throughout nature, magnifies its claims to every species of investigation. Davy detected it in six marine plants (Phil. Trans. 1814), and Dr. Fyfe added the sponge (Ed. Phil. Journ.), since which it has been shown to be present in several marine animals, in various European and American saline springs, (United States Dispensatory), in ores of silver and lead from Mexico, and by M. Mentzel in a zinc-ore from Upper Silesia (Feruss. Bull. 1828.) Lastly, two phænogamous plants of the genera Salsola and Alöe, growing in the interior of America, have been found to contain iodine." (P. 14.)

But a most remarkable fact, and which may be said to furnish an additional link in the chain connecting organic and inorganic matter, has escaped our author's attention, namely, that an insect has been found near Ascoli in Italy, which Savi has described under the name of the *Julus fœtidissimus,* containing iodine. The animal emits when disturbed a yellow fluid strongly smelling of iodine, and which immediately strikes the characteristic violet colour with starch.*

There is a schism among medical writers regarding the manner in which the agency of iodine on the living body is affected by chemical union. Some are of opinion that iodine and its compounds all follow essentially the same line of action, while others maintain that the compounds in question " combine the properties of the substance united to the iodine with those of the substance, and operate accordingly."

" As these theories cannot be both true together, the writer imagined, correctly or not, that the path intended for him to pursue was that of making original investigations, with a view of determining whether either, or which of them, had its foundation in nature. Unfortunately, he considers that his results do not distinctly warrant him in adopting the conclusion of either party." (P. 11.)

It would be a difficult matter to reconcile the statements promulgated by practitioners of repute as to the excessive doses of iodine which can be borne without detriment to the system, in opposition to physiological experiments, did we not hold in mind the fact that this element in certain officinal preparations is prone to spontaneous changes, and liable to modification or decomposition from various substances it may encounter in the first passages. Thus with albumen it forms a compound insoluble in water and wine; beer or cyder convert it into iodic and hydriodic acids.

" If there are facts on one side which seem to shew that iodine is devoid of energy, it certainly would not be easy to draw the same inference from those we have just mentioned, more particularly the results of Orfila. The latter are likewise more in accordance with general experience. While examples of inertness, therefore, are comparatively rare, they constitute no rule, but must rather be regarded as exceptions to a general law; nor, in the present instance, do the exceptions appear to be entirely inexplicable, by reference to the tendency of iron to combine with various constituents of our common nutriment." (P. 27.)

Roulin has attempted to shew that iodine, chlorine, and bromine are identical in their therapeutical effects. This notion, however, is based rather on imperfect truth than fundamental error. Their effects are congenerous in as far as they are all powerfully irritant and corrosive. In the fatal cases recorded by Zink and Jahn, the iodine exerted a marked irritant action on the digestive apparatus.

Dr. Cogswell next proceeds to describe the injurious effects of iodine

* Magazin für Pharmacie, Bd. xxi. p. 31.

on the several systems of the body. Thus, in the alimentary canal it is capable, as above mentioned, of producing notable disorder in the primæ viæ. Touching the nervous system, we are told, that, "generally speaking, when iodine begins to disagree with a patient, one of the foremost among the bad symptoms is a sensation of giddiness or headach, or both united." Hence great caution is enjoined in the use of iodine by Sir Benjamin Brodie, who asserts that it has been known to occasion paralysis. We ourselves have repeatedly seen affections of the nervous system approaching paralysis produced by it, namely, extreme irritability, trembling, and jactitation. These effects were of long duration and were accompanied by general emaciation. The whole tract of the air passages seems to be under the influence of iodine, as is proved by its giving rise to hæmoptysis and other symptoms of thoracic irritation. On the salivary glands it has a peculiar action, as indicated by its both determining and arresting ptyalism. In the liver and spleen it has been found to occasion inflammation. Udall expressly states, in connexion with its producing hepatic irritation, that patients recently cured of ague relapsed so soon as iodine came to be administered to them.* In reference to the generative system, it may be averred that iodine is a powerful stimulant to the uterus; it has determined atrophy of the mammæ and testes. Lugol believes it to be a diuretic, and Udall confirms this opinion. Dr. Cogswell has failed to notice the singular circumstance mentioned by Hoffmann and Udall of its darkening the urine and causing the precipitation of a black deposit. The last-named observer instances the case of a person in whom this phenomenon occurred, who had within a space of six days about four grains and a half of iodine and double that quantity of hydriodate of potash, and which lasted for several months, although he had only taken the medicine during ten days.†

On the skin it produces sometimes a dingy smoked appearance, at other times gives rise to erythema and pustules. Dr. Cartwright, of Natchez, has mentioned a singular phenomenon incident to the application of ioduretted ointments; namely, their causing a total loss of sensation in that part of the integuments on which the friction is made, and sometimes extending the paralyzing influence to those parts which are supplied by the same nerves of sensation.‡ It is not only a stimulant to the absorbent system, but in some instances appears to exercise a modified influence over the nervous or vital actions of a diseased surface, as pointed out by Mr. Key. Udall entertains the opinion that iodine produces emaciation in virtue of its powerful incitement of the absorbent system. Hence the emaciation proceeds faster in children than in adults. Dr. Gräfe, of Berlin, employed iodine as an "emaciating agent" with complete success in a case of *polysarcia*, after having used active depletory means with partial relief only.§

To the question, whether iodine be a cumulative medicine, Dr. Cogswell has not been able to give a very satisfactory answer. The experimental enquiry instituted for this purpose turned out to be both complicated and

* Diss. de Effect. Iodinii Havniæ, 1833. † Op. cit.

‡ American Med. Recorder, No. xliv.

§ Hobson on Iodine, in Transactions of the Medical Society of the State of New York. Vol. II. p. 299.

difficult. His experiments on the urine of individuals taking the medicine led to approximative results only.

"I think we can deduce from the above results that iodine taken, as it has been usually prescribed, in the form of tincture mixed with water, becomes less readily absorbed, and probably remains longer in the bowels, than in the other forms already mentioned. Sometimes the *iodine* made a quick passage to the kidneys, but in the greater number of cases its appearance in their secretion was exceedingly tardy, unless indeed it made its escape in the intervals of testing the urine, which does not appear probable. It even varied in the same individual at different periods, appearing, departing, and reappearing without any evident cause. The same was not the case with the iodide of potassium, and perhaps of iron; the former is even noted for passing rapidly out of the system." (P. 62.)

Were we asked what are the chronic ailments in which iodine has been employed, we should answer, that there are few, if any, in which it has not. When judiciously administered, it improves the appetite, strengthens the patient, proves diuretic; is, in short, a powerful *energizer* of the functions of nutrition. Hence its utility in struma; in atonic affections of the uterus; in secondary lues complicated with scrofula, where there are diminution of strength and morbid irritability; in hydropic effusions; in varicose veins; in visceral enlargements, &c. A remarkable example of the curative effects of iodine in Angina pectoris occurred in the person of Dr. Oliver, of Massachusetts. He took the medicine dissolved in alcohol, of the strength of twenty grains to the ounce, thrice in the day, beginning with six drops, and gradually increasing it to sixteen or twenty. "I think," says Dr. O., in a letter to Dr. Silliman, "that I derived as much benefit from the iodine in a fortnight as I had from the solution of sublimate in eleven months, and indeed I may say much more."*

The iodic preparation now generally employed is the hydriodate of potash; although, according to our author, the combination of this with iodine is the form most generally approved of, whether for internal or external use. We should be here in a like perplexity as we were in respect to iodine, as to the quantity of the hydriodate which may be exhibited with impunity, were we not aware that the salt of commerce is oftentimes very impure. This source of error, which appears to have misled some of our most enlightened physicians, may be ascribed either to spontaneous conversion of the iodide of potassium into carbonate of potash, through atmospheric influence, or to its adulteration with the same drug by fraudulent venders.

"For an easy method of detecting the sophistication with carbonate of potash, I am indebted to Dr. Traill, who practised it as early as 1826. Tincture of iodine, added to the suspected compound, loses its colour if the carbonate be present. Mr. Pereira proposes to immerse one of the crystals in a little lime-water, when the presence of carbonic acid will be indicated by the fluid being milky. To test for common salt, the same pharmacologist describes a process founded on the capacity of ammonia to dissolve the chloride, but not the iodide of silver. On adding nitrate of silver to the suspected sample, any carbonates, chlorides, and iodides are thrown down; digesting the precipitate in ammonia, you take up only the carbonate and chloride; and, on adding nitric acid to this solution, the carbonate is converted into nitrate, and the chloride is precipitated." (P. 87.)

It is fully established that the hydriodate of potash may, and does

* Hobson on Iodine, in lib. cit., p. 299.

exert a powerfully irritant action on the digestive canal. It may likewise induce deleterious effects on the nervous system. It has been known to give rise to coryza in the air-passages, pustules on the skin, ptyalism, diuresis. As to its medicinal effects, it seems to be less efficient in goitre than simple iodine. In scrofula, though powerful, it takes longer time to manifest its action; but, again, there is less danger in continuing it a sufficient length of time. Of its utility in phthisis we believe that no positive proof is on record. It has failed in nervous complaints, where iodine has been of remarkable benefit. We concur with what we ourselves have heard expressed by our most distinguished hospital physicians and surgeons, that the dose of hydriodate of potash ought not to exceed five grains. The above quantity, repeated at suitable intervals, and continued for a period of weeks, will prove advantageous wherever the remedy is indicated. When we go beyond this measure, we are apt to induce iodism, or, at all events, gastric derangement.

The iodide of sulphur has been employed successfully for the external treatment of skin diseases; as a resolvent of the tubercles in lupus and acne indurata, in inveterate lepra, in impetigo figurata, and in tinea.

Two compounds of iodine with carbon are described, a protiodide and a sesqui-iodide. Both are endowed with intensely irritant qualities. The sesqui-iodide seems to be allied to the toxicological group of Strychnos and Brucea; inasmuch as its chief influence appears directed to the spinal marrow, causing convulsions and difficult breathing, but no cerebral disturbance. The protiodide has been used with advantage as an outward application to lepra and porrigo, in an ointment containing half a drachm of the powder to six drachms of simple cerate.

In the compounds of iodine with the electro-positive elements, the energy of the latter seems to preponderate. Thus, the iodide of iron, administered in large doses, produces effects on the system analogous to those of the sulphate. Dr. Cogswell has ascertained by experiment,

"1. That it (iodide of iron) acts as a local stimulant, with the power of effecting peculiar disorganization. 2. That its action is more particularly directed to the tract of the alimentary canal. In these respects, although it may bear some faint degree of resemblance to iodine and hydriodate of potash, yet the details are far from corresponding with much accuracy." (P. 132.)

A little further on, the author remarks, "The iodide of iron seems rather to affect the character of a ferruginous than that of an iodinous compound." One great defect of the hydriodate of iron, and which must militate against its employment in practice, is its easy decomposition.

Two iodides of mercury have been introduced into practice, the protiodide and the biniodide. The former would seem to be a compound of no inconsiderable energy. It has chiefly been exhibited in syphilitic affections implicating the cutaneous tissue. The biniodide is, without doubt, a most powerful irritant. Lugol considers it nearly as escharotic as corrosive sublimate. Dr. Cogswell is not acquainted with any specific example of its causing salivation. We, however, have witnessed this result follow the continued application of a minute quantity, in the form of ointment, to ulcers in the legs.

In terminating our analysis of this work, we subjoin the summary of the author's conclusions from the facts collated by him.

"1. That iodine and hydriodate of potash act very much in the same way, but that there is still a difference, not merely in point of power, but of specific properties. 2. That whatever be the proper action of the iodide of sulphur, its facility of decomposition gives it a resemblance to iodine. 3. That the iodides of carbon, so far as examined, have an action peculiar to themselves. 4. That, in those metallic iodides which were submitted to examination, the preponderance of power is on the side of the bases." (P. 167.)

---

